# PDI Family Members as Guides for Client Folding and Assembly

**DOI:** 10.3390/ijms21249351

**Published:** 2020-12-08

**Authors:** Shingo Kanemura, Motonori Matsusaki, Kenji Inaba, Masaki Okumura

**Affiliations:** 1 School of Science and Technology, Kwansei Gakuin University, 2-1 Gakuen, Sanda, Hyogo 669-1337, Japan; shingo.kanemura@kwansei.ac.jp; 2 Institute of Multidisciplinary Research for Advanced Materials, Tohoku University, 2-1-1 Katahira, Aoba-ku, Sendai, Miyagi 980-8577, Japan; matsusaki@tohoku.ac.jp (M.M.); kenji.inaba.a1@tohoku.ac.jp (K.I.); 3 Frontier Research Institute for Interdisciplinary Sciences, Tohoku University, 6-3 Aramakiaza Aoba, Aoba-ku, Sendai, Miyagi 980-8578, Japan

**Keywords:** endoplasmic reticulum (ER), protein disulfide isomerase (PDI) family, redox, disulfide, protein folding, assembly, disassembly

## Abstract

Complicated and sophisticated protein homeostasis (proteostasis) networks in the endoplasmic reticulum (ER), comprising disulfide catalysts, molecular chaperones, and their regulators, help to maintain cell viability. Newly synthesized proteins inserted into the ER need to fold and assemble into unique native structures to fulfill their physiological functions, and this is assisted by protein disulfide isomerase (PDI) family. Herein, we focus on recent advances in understanding the detailed mechanisms of PDI family members as guides for client folding and assembly to ensure the efficient production of secretory proteins.

## 1. Introduction

A typical mammalian cell expresses approximately 20,000 different proteins, more than 70% of which contain multiple domains [1]. These proteins must adopt specific three-dimensional structures to perform their biological functions. Although numerous studies on single-domain protein folding have been reported, there are relatively few studies on how interactions between subunits/domains affect protein folding and assembly to obtain native quaternary structures [1]. Tubulin, a heterodimer composed of α and β subunits [2], has been employed as a model for protein folding and assembly research. The eukaryotic chaperonin, tailless complex polypeptide 1 ring complex (TRiC)/chaperonin-containing tailless complex polypeptide 1 (CCT), an ATP-dependent chaperone in the cytosol, is required to ensure native folding and subunit assembly of tubulin [3]. TRiC also prevents aggregation and toxicity of proteins linked to amyloid neurodegenerative disorders [4]. Therefore, cell viability is maintained by chaperone networks that control protein folding, assembly, antiaggregation, disaggregation, and degradation. Over several decades, many researchers have gained substantial insight into protein homeostasis (proteostasis) networks in the cytosol [5].

Meanwhile, approximately one-third of all newly synthesized proteins such as antibodies and insulin are inserted into the endoplasmic reticulum (ER). Disulfide bond formation can drive folding, inhibit unfolding [6,7,8], and stabilize oligomeric protein assemblies [9]. The newly synthesized proteins cotranslationally inserted into the ER are structurally immature, and oxidative folding is completed while they are protected against aggregation in the crowded ER environment, after which they are transported to the Golgi apparatus [10]. The oxidized and reduced glutathione ratio in the ER ranges from 1:1 to 1:3, and this redox environment helps to promote oxidative folding reactions [11]. The protein disulfide isomerase (PDI) family, of which there are more than 20 types present in the ER, forms a sophisticated proteostasis network in this organelle [12,13,14,15]. These PDI family proteins target a wide range of substrates such as antibodies and major histocompatibility complex (MHC) class-I [16,17]. This review summarizes recent findings and the underlying mechanism by which PDI family members catalyze client folding and assembly to maintain proteostasis in the ER.

## 2. Structural Insight into PDI-Regulated Client Assembly

### 2.1. Redox-Dependent Conformational Changes of PDI

**Figure 1:**
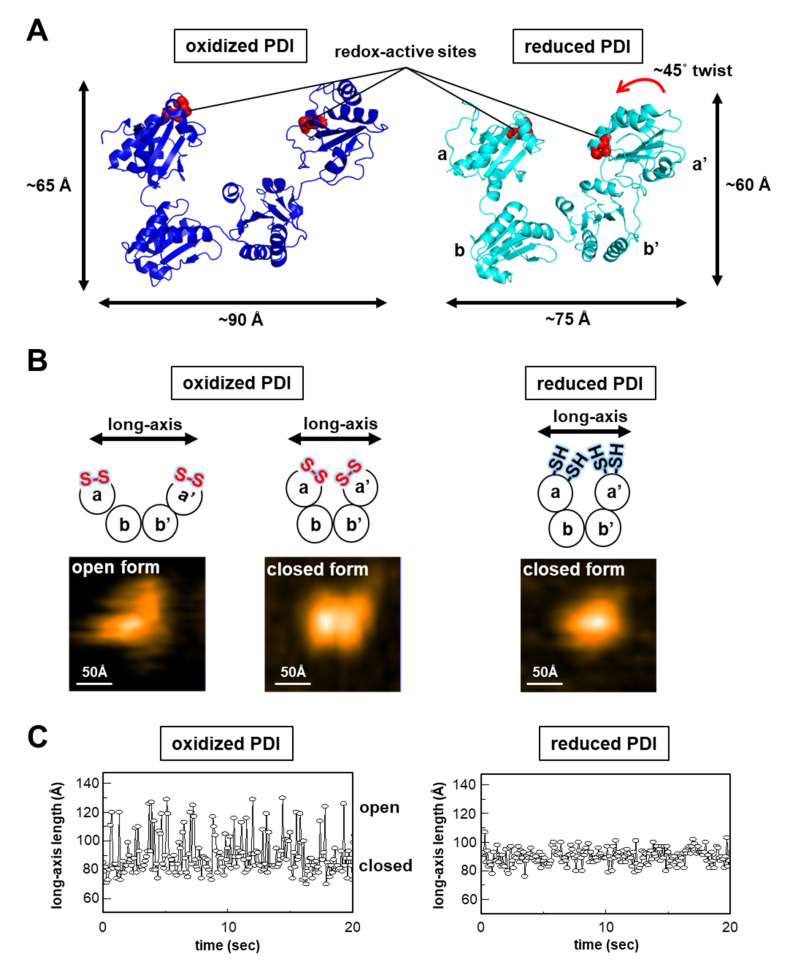
Redox-regulated dynamic nature of the *human* protein disulfide isomerase (PDI) molecule. (**A**) Left and right panels show crystal structures of oxidized PDI (Protein Data Bank (PDB) code: 4EL1) and reduced PDI (PDB code: 4EKZ) from *human*, respectively. Redox-active sites of oxidized and reduced PDI are indicated by red sphere. (**B**) High-speed atomic force microscopy (HS-AFM) images (lower; scan area 200 × 200 Å^2^; scale bar 50 Å) of oxidized (left) and reduced (right) forms of *human* PDI. (**C**) Time trace of the long-axis length for oxidized (left) and reduced (right) forms of *human* PDI.

PDI (also known as PDIA1 and P4HB) is identified as an enzyme that promotes oxidative folding [18,19,20] and acts as a molecular chaperone [21]. PDI consists of four thioredoxin (Trx)-like domains named **a**, **b**, **b’**, and **a’**, which form a compact U-shape [22] (Figure 1A). Both **a** and **a’** domains contain a Cys-X-X-Cys motif as a redox-active site essential for thiol-disulfide exchange reactions with substrate proteins. To recruit clients, the *human* PDI **b’** domain provides the principal substrate-binding sites based primarily on a hydrophobic pocket including Phe240, Phe249, and Phe304 [23,24]. Dynamic light scattering and small-angle X-ray scattering (SAXS) analyses indicate that binding of the inhibitor bisphenol-A to the substrate-binding sites of PDI **b’** induces significant rearrangement of its N-terminal Trx-like domain, resulting in a more compact overall structure of PDI [25]. This conformational change leads to closure of the substrate-binding pocket in the **b’** domain, preventing PDI from binding to other clients. In addition to conformational changes caused by substrate binding to the **b’** domain, the redox state of the active site, especially in the PDI **a’** domain, undergoes striking conformational changes that affect substrate selectivity [22]. Redox-dependent client binding/release by PDI has been reported for several substrates, including cholera toxin, antigen peptides, and the α-subunit of prolyl-4-hydroxylase (P4-H) [26,27,28]. Crystal structures of reduced and oxidized PDI from *human* revealed redox-dependent conformational switching [22]. In reduced PDI, **a**, **b**, and **b’** domains are arranged on the same plane, and only the **a’** domain twists outward by ~45° (Figure 1A, right). By contrast, in the oxidized state, all four domains are arranged on the same plane (Figure 1A, left). The distance between the two redox-active sites in the **a** and **a’** domains of the oxidized form is shifted by more than 12.7 Å relative to the reduced form, resulting in an increase in the inner space within the U-shaped domain arrangement, which is required for client binding/release. Indeed, oxidized PDI has higher chaperone activity than reduced PDI [29]. This is because the hydrophobic inner surface inside the U-shaped PDI conformation is more exposed to solvent, allowing large substrates to gain access to the expanded interior space. Regarding redox-dependent conformational changes, a cation-π interaction between residues Arg300 in the **b’** domain and Trp396 in the **a’** domain plays a pivotal role in conformational switching; disulfide bond formation between Cys397 and Cys400 of the **a′** domain disrupts cation-π interactions between the guanidinium group of Arg300 in the **b′** domain and the indole ring of Trp396 in the **a′** domain, resulting in an open state in the oxidized form [22]. Of note, redox-dependent conformational changes play a role in modulating the function of mammalian PDI, more so than in the *Saccharomyces cerevisiae* and *Thermophilic fungus* PDI enzymes, because Arg300, an essential residue for redox-driven conformational switching, is highly conserved among mammalian PDI enzymes but lacking in the *S. cerevisiae* [30] and *T. fungus* proteins [31].

In agreement with structural information obtained from crystallographic analyses, our SAXS measurements revealed redox-dependent conformational changes for *human* PDI in solution [32]. The radius of gyration (*R*_g_) was estimated to be 35.8 ± 0.2 Å and 37.2 ± 0.2 Å for the reduced and oxidized forms, respectively. The largest *r* values (*D*_max_) were estimated to be 126 Å for the reduced form and 138 Å for the oxidized form, indicating that the oxidized form has a more extended conformation than the reduced form in solution. Intriguingly, single-molecule observations using high-speed atomic force microscopy (HS-AFM) provided new insight into the redox-dependent conformational changes of *human* PDI. Oxidized PDI exists as two populations, with long-axis lengths of 82 ± 9 Å for the closed form and 111 ± 17 Å for the open form, while reduced PDI exists as a single population with long-axis lengths of 86 ± 12 Å for the closed form [32] (Figure 1B). Time-resolved single-molecule analysis by HS-AFM further demonstrated that oxidized PDI is in rapid equilibrium between open and closed conformations, whereas reduced PDI is maintained in the closed state [32] (Figure 1C). These results imply that redox-dependent conformational dynamics of *human* PDI is important for client binding/release.

### 2.2. Unfolded Substrate-Induced PDI Dimerization

**Figure 2:**
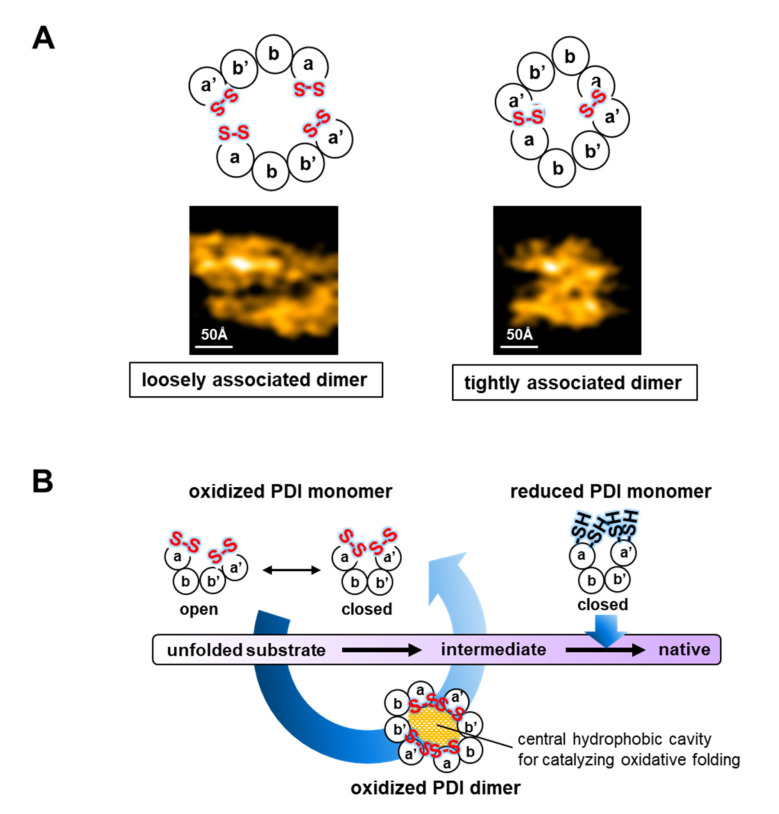
The dimeric PDI-mediated oxidative protein folding process. (**A**) Schematic structures (upper) and HS-AFM images (lower; scan area 200 × 200 Å^2^; scale bar 50 Å) of loosely (left) and tightly associated dimers (right) of *human* PDI. (**B**) Schematic diagram of redox-dependent conformational changes and substrate-induced PDI dimerization during the catalysis of oxidative protein folding.

The redox-regulated conformational switch in PDI appears to be beneficial for the binding/release of various clients with different structures along client folding pathways; oxidized PDI enables the introduction of disulfide bonds in unstructured substrates during the initial folding step, whereas reduced PDI presumably traps partially structured folding intermediates and rearranges non-native disulfide bonds. PDI has several binding sites in the central redox-inactive **b** and **b’** domains with affinity sufficient to enable the binding/release of clients along folding pathways. Indeed, surface plasmon resonance experiments demonstrated that the affinity decreases along the folding pathway; *K*_D_ values decrease from native (394 μM) to partially folded (~14 μM) and unfolded (~3 μM) states [33]. This suggests that both the client folding status and the redox-dependent rearrangement of the PDI domains determine client binding/release. Strikingly, HS-AFM images revealed that in the presence of constitutively reduced and unfolded model clients, bovine pancreatic trypsin inhibitor (BPTI) and ribonuclease A (RNase A), more than half of oxidized PDI molecules (~60%) form a face-to-face homodimer that creates a central hydrophobic cavity with multiple redox-active sites for efficient oxidative protein folding [32] (Figure 2A). In the presence of partially structured folding intermediate of BPTI, which is not able to fold, and native BPTI, the fractions of oxidized PDI dimer are 26.6% and 12%, respectively. In contrast with oxidized PDI, reduced PDI forms only a minor fraction of homodimer (~15%), even in the presence of excess amounts of constitutively reduced and unfolded BPTI as a model substrate [32]. Thus, unfolded substrate-induced dimerization is only observed with oxidized PDI, suggesting a relationship between redox-dependent conformational dynamics and PDI dimerization.

The central hydrophobic cavity generated by PDI dimers is diverse in terms of lifetime and shape. In the presence of foldable reduced and denatured BPTI, assembly into dimers and disassembly into monomers are repeatedly observed with a period of ~1 s according to HS-AFM analysis [32]. On the other hand, constitutively reduced/unfolded clients generate noncovalent PDI dimers over ~20 s. In line with this, the PDI dimer induced by RNase A, the folding of which is slower and more complex than that of BPTI [34], lasts longer than the BPTI-induced dimers, and undergoes repeated expansion and contraction. Regarding the shape of the PDI dimer, statistical analyses using the Akaike information criterion revealed that this dimer exists as two populations in the presence of unfolded client, with long-axis lengths of 135 ± 18 Å for a loosely associated form, and 95 ± 11 Å for a tightly associated form [32] (Figure 2A). On the other hand, the population of folding intermediate-induced PDI dimers was best fitted by a single-state model with a long-axis length of 116 ± 20 Å. Such transformable PDI dimers suggest that the interactions between two PDI molecules are weak and transient, depending on the folding states of clients.

Regarding the restriction of cavity size and client capacity, multiple PDI dimers bind to large substrates, such as the 90 kDa plasminogen and the 87 kDa laminin-511, suggesting that PDI dimers bind to local conformations of large substrates to allow oxidative subunit/domain folding simultaneously at multiple sites [32]. Collectively, PDI regulates conformational dynamics and oligomeric states in accordance with its own redox state and the configurations or folding states of substrates to effectively guide proper oxidative protein folding (Figure 2B).

### 2.3. PDI-Mediated Folding and Assembly of Human Chorionic Gonadotropin (hCG)

**Figure 3:**
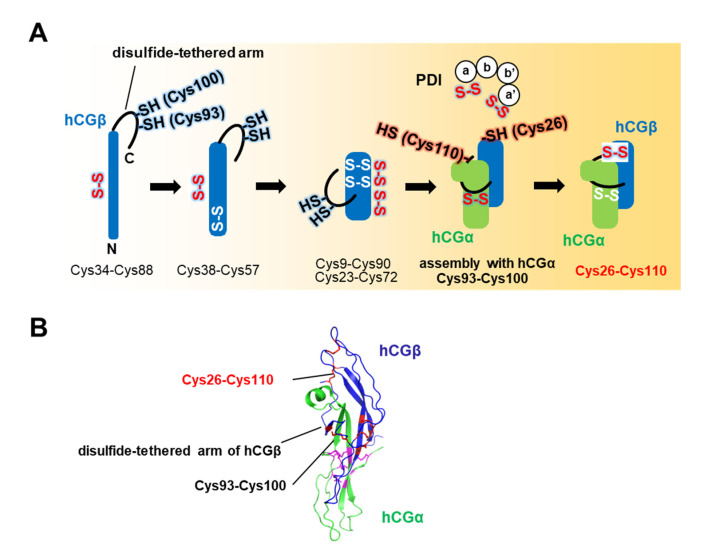
PDI-assisted *human* chorionic gonadotropin (hCG) folding and assembly. (**A**) Schematic diagram of the PDI-assisted folding and assembly pathway of hCG. After hCG assembly by PDI, Cys93-Cys100 on the arm of hCGβ and Cys26-Cys110 disulfide bonds form to embrace hCGα. (**B**) Crystal structure of the hCG complex (PDB code: 1HCN). hCGα and hCGβ are colored green and blue, respectively. Disulfide bonds of hCGα and hCGβ are represented by magenta and red sticks, respectively. In particular, hCGα is recognized by a loose loop flanking a Cys93-Cys100/Cys26-Cys110 disulfide bond that positions hCGβ.

PDI functions not only as a catalyst of oxidative folding, but also as an assistant of assembly (Figure 3A). *Human* chorionic gonadotropin (hCG) is a placental hormone that stimulates secretion of the pregnancy-sustaining steroid progesterone, and promotes the maintenance of the corpus luteum during the beginning of pregnancy [35]. hCG is a non-covalently-linked heterodimeric glycoprotein hormone composed of an α-subunit (hCGα) and a β-subunit (hCGβ), and it follows a unique folding and assembly pathway. In the structure of hCG, hCGα is embraced by the disulfide-tethered arm of hCGβ [36] (Figure 3B). Detailed analyses of hCGβ oxidative folding and assembly with hCGα demonstrated that the disulfide bonds in hCGβ are formed in the order Cys34-Cys88, Cys38-Cys57, and Cys9-Cys90/Cys23-Cys72 [37,38,39,40] (Figure 3A). In the final assembly step, hCGα is recognized by a loose arm containing Cys93-Cys100/Cys26-Cys110 disulfide bonds of hCGβ. Cleavage of these disulfide bonds then initiates the assembly of hCGα and hCGβ without disrupting the overall hCGβ structure [37]. After assembly, the disulfide bonds of hCGβ are re-crosslinked in the order Cys93-Cys100 and Cys26-Cys110. Remarkably, PDI acts on the kinetically trapped folding intermediate of hCGβ lacking disulfide bonds Cys93-Cys100 and Cys26-Cys110 during the final step, and then accelerates the assembly of hCG subunits without changing the order of disulfide bond formation [37] (Figure 3A). Thus, PDI catalyzes disulfide introduction/exchange reactions of kinetically trapped on-pathway intermediates to prompt both the folding of individual polypeptides [32,34,41] and subunit assembly [37].

## 3. Molecular Basis of Endoplasmic Reticulum Resident Protein 57 (ERp57)-Assisted Client Assembly

### 3.1. Client Recognition Mechanism by ERp57 in Concert with Calnexin (CNX), Calreticulin (CRT), and ERp27

**Figure 4:**
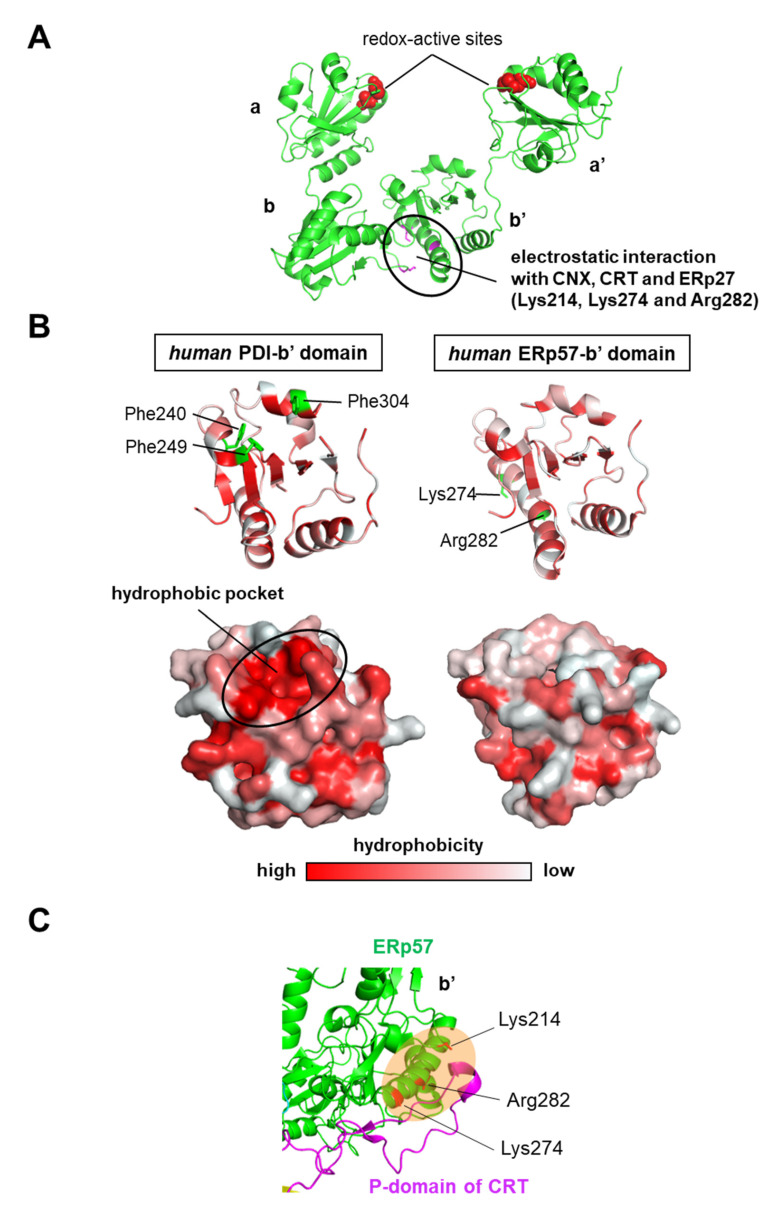
Client recognition mechanism by *human* endoplasmic reticulum resident protein 57 (ERp57). (**A**) Crystal structure of ERp57 (PDB code: 3F8U). Redox-active sites are shown as red spheres. The electrostatic interaction site of the ERp57 **b’** domain with concert with calnexin (CNX), calreticulin (CRT), and ERp27 is shown as a black circle. Electrostatic interaction residues Lys214, Lys274, and Arg282 are shown as magenta sticks. (**B**) Upper panels show crystal structures of **b’** domains in oxidized *human* PDI (left, PDB code: 4EL1) and *human* ERp57 (right, PDB code: 3F8U). The *human* PDI **b’** domain provides the principal substrate binding sites based primarily on a hydrophobic pocket including Phe240, Phe249, and Phe304. The *human* ERp57 **b’** domain does not have a hydrophobic pocket, but positively charged residues Lys274 and Arg282, which are important for the interaction with CNX, CRT, and ERp27, are located on the outer surface of the ERp57 **b’** domain. Lower panels show surface representations of oxidized PDI (left) and ERp57 **b’** domains (right). The color is coded ranging from hydrophobic (red) to hydrophilic (white) according to the normalized consensus hydrophobicity scale of the surface-exposed residues. The hydrophobic pocket of the oxidized PDI **b’** domain is shown as a black circle. (**C**) Electrostatic interaction site between the positively charged region of the **b’** domain in ERp57 and the negatively charged region of the P-domain in CRT (PDB code: 6ENY).

*Human* ERp57 (ERp60 and PDIA3) is another PDI family member with a PDI-like U-shaped structure composed of **a**, **b**, **b’**, and **a’** domains [42]. ERp57 catalyzes the oxidative folding of glycoproteins in concert with the lectin chaperones calnexin (CNX) and calreticulin (CRT) as the client recruiter [43,44,45,46]. The first three-dimensional structure of ERp57 was determined as an ERp57-tapasin complex by crystallographic analysis, and its domain orientation is similar to that of PDI [42] (Figure 4A). Both **a** and **a’** domains contain a Cys-X-X-Cys motif as a redox-active site that is essential for thiol-disulfide exchange reactions with substrate proteins. The *human* ERp57 **b’** domain does not have a hydrophobic pocket (Figure 4B), but positively charged residues Lys214, Lys274, and Arg282 are located on the outer surface of the ERp57 **b’** domain, and these engage in electrostatic interactions with the negatively charged tip of the arm-like P-domain in CNX and CRT, and the negatively charged loop of the ERp27 **b’** domain [47,48,49,50,51] (Figure 4C). Of note, nonpolar residues located in the substrate-binding pocket in the PDI **b’** domain are substituted by polar residues in the ERp57 **b’** domain [42], suggesting significant differences in the client recognition mechanism. Despite the similarity in overall domain arrangement, ERp57 is much less capable of introducing native disulfide bonds into reduced and denatured RNase A than PDI [52]. Unlike PDI, *human* ERp57 cannot control redox-dependent conformations and client recruitment along the client folding pathway because it lacks the amino acid residue corresponding to Arg300 of *human* PDI [42], an essential residue for redox-driven conformational switching, and the ability to recruit clients by itself. Taken together, differences in client recognition between PDI and ERp57 with CNX/CRT allow the control of the folding status of a wide range of clients, and different physiological roles may facilitate more accurate monitoring of proteostasis.

### 3.2. ERp57-Assisted Folding and Assembly of Neonatal Fc Receptor (FcRn) and Fibrinogen

**Figure 5:**
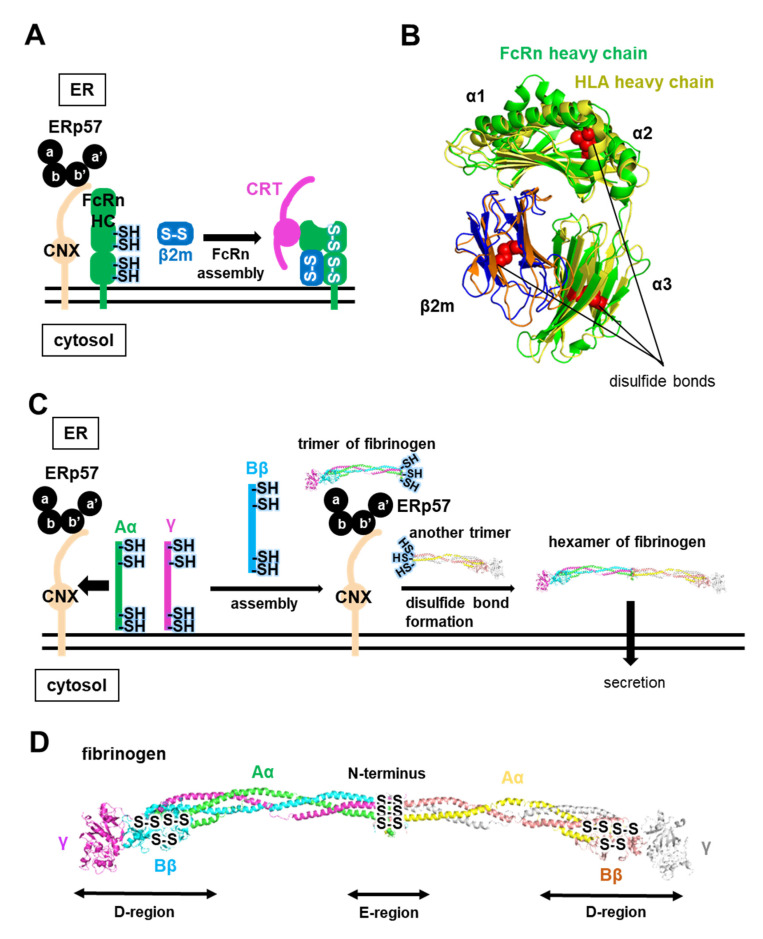
ERp57-CNX interplay determines subunit assembly. (**A**) Schematic diagram of the folding and assembly pathway of neonatal Fc receptor (FcRn) assisted by ERp57 with CNX/CRT. CNX with ERp57 recognizes FcRn-heavy chain (HC) through monoglucosylated N-linked glycans. FcRn-HC and β2m then assemble, and assembled FcRn binds CRT. (**B**) Superimposition of the crystal structures of FcRn (PDB code: 6C97) and *human* leukocyte antigen (HLA) (PDB code: 6ENY) heterodimers. FcRn and HLA heavy chain are colored green and yellow, respectively. Disulfide bonds are shown as red spheres. (**C**) Schematic diagram of the folding and assembly pathway of ERp57-assisted fibrinogen in concert with CNX. CNX with ERp57 recognizes the fibrinogen αγ complex through monoglucosylated N-linked glycans, and the newly synthesized fibrinogen β chain is then integrated into the αγ complex. A fibrinogen trimer is formed and handed over to ERp57 from CNX. ERp57 finally facilitates the integration of the two trimers into the hexamer. (**D**) Crystal structure of fibrinogen (PDB code: 3GHG). Aα-chain, Bβ-chain, and γ-chain are colored green, cyan, and magenta, respectively.

It is reported that ERp57 assists the assembly of neonatal Fc receptor (FcRn), an MHC class-I-related receptor. FcRn transfers maternal immunoglobulin G (IgG) from mother to young via the neonatal intestine, and regulates serum IgG levels to establish neonatal immunity [53]. Regarding the proper folding and assembly of FcRn, FcRn reportedly interacts with certain chaperones. ERp57 and CNX bind to the FcRn heavy chain before subunit assembly with the light chain, but CRT only associates with assembled FcRn [54] (Figure 5A). Thus, the ERp57-CNX system plays an obligatory role for FcRn assembly, as is the case for *human* MHC class-I (*human* leukocyte antigen, HLA) assembly (see Section 5.2). Similar to the subunit assembly of HLA, FcRn is composed of an N-glycosylated heavy chain containing two disulfide bonds that is non-covalently attached to a β_2_-microguloblin (β2m) light chain containing one disulfide bond [55] (Figure 5B). This heavy chain consists of α1, α2 and α3 external domains that are anchored to the cell surface by a short transmembrane domain and a cytoplasmic tail, like the HLA heavy chain [56,57,58]. However, unlike HLA, tapasin and transporter associated with antigen processing (TAP) are not involved in FcRn assembly in the ER, suggesting that FcRn does not form a supramolecular complex such as the peptide loading complex (PLC) during substrate loading.

ERp57 also participates in the assembly of fibrinogen, a precursor protein of fibrin, a glycoprotein found in the blood plasma of all vertebrates [59] (Figure 5C). Several lines of evidence indicate that ER-resident chaperones such as CNX, CRT, and ERp57 are involved in its assembly [60,61] (Figure 5C). Mature fibrinogen consists of a covalently linked hexamer composed of two sets of symmetrical trimers (α, β and γ), and one set is divided into one central E-region domain and two additional D-region domains [62,63] (Figure 5D). The E-region and D-region are connected by an α-helical coiled-coil segment composed of the three chains. Fibrinogen contains 29 intra- or intermolecular disulfide bonds, but no free thiols. Of note, these disulfide bonds are involved in intrasubunit assembly; the coiled-coil region is flanked at both ends by intermolecular disulfide bonds, and the central E domain contains three symmetrical disulfide bonds at the N-terminal region [62,63] (Figure 5D). The stepwise disulfide-dependent assembly of the fibrinogen hexamer proceeds from two-chain complexes to three-chain half-molecules, which are finally coupled together to form six-chain fibrinogen [64,65]. CNX recognizes the fibrinogen αγ complex through monoglucosylated N-linked glycans, and ERp57 transiently binds to the fibrinogen trimer during assembly via CNX. ERp57 finally facilitates the integration of the two trimers into the hexamer via the formation of three symmetrical disulfide bonds at the N-terminal region (Figure 5C). The kinetic rate of fibrinogen assembly is controlled by ERp57 in HepG2 cells [66]. Thus, ERp57 is involved in kinetic control of stepwise fibrinogen assembly in concert with CNX.

## 4. Endoplasmic Reticulum DNA J-Domain-Containing Protein 5 (ERdj5)-Catalyzed Oxidative Folding and Reductive Unfolding

### 4.1. Structural Features of ERdj5

**Figure 6:**
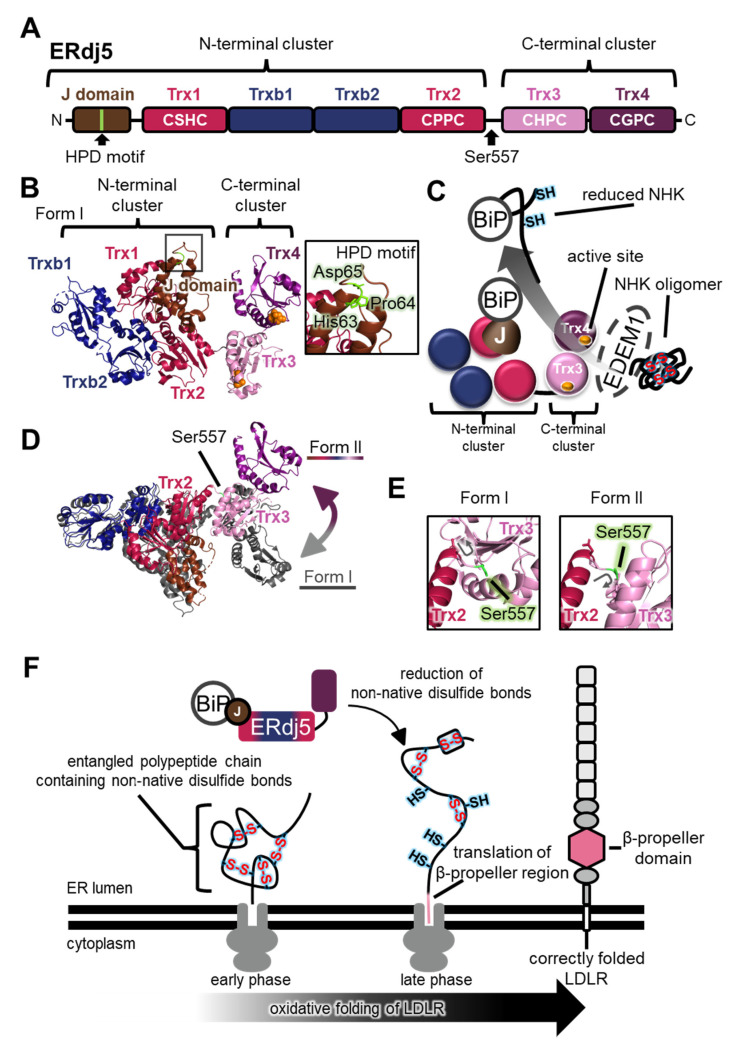
The highly dynamic nature of endoplasmic reticulum DNA J-domain-containing protein 5 (ERdj5) plays a key role in functional interplay with substrates. (**A**) Schematic representation of the ERdj5 domain structure. The position of the His-Pro-Asp (HPD) motif that is important for association with BiP and the Ser557 residue that is critical for linker flexibility are indicated. (**B**) (Left) Crystal structures of ERdj5 form I (each domain colored as in (**A**); PDB code: 5AYK). Redox-active sites in Trx3 and Trx4 are shown as orange spheres. (Right) Close-up view of the HPD motif in the J-domain is indicated. (**C**) Handling of the endoplasmic reticulum (ER)-associated degradation (ERAD) substrate null Hong Kong (NHK) oligomer by ERdj5. EDEM1 recognizes disulfide-linked NHK oligomers and associates with the C-terminal cluster of ERdj5 [68]. Active sites in Trx3 and Trx4 reduce the intermolecular disulfide bonds in NHK oligomer [68]. Finally, reduced NHK is delivered to BiP on the J-domain of ERdj5, and BiP dissociates from ERdj5 to recruit reduced NHK for retrograde translocation. (**D**) Superimposition of crystal structures of form I (gray; PDB code: 5AYK) and form II (each domain colored as in (**A**); PDB code: 5AYL). The C-terminal cluster is shifted upward relative to the N-terminal cluster in form II, suggesting movement between these clusters, as indicated by the two-way arrow. Ser557 in the flexible linker of form II is indicated. (**E**) Different orientations of Ser557 in form I and form II (each domain colored as in (**A**)). Ser557 is colored in light green. Gray arrows indicate the orientation of the main chain between Trx2 and Trx3 in form I and form II, respectively. (**F**) Schematic model of co-translational oxidative folding of LDLR and reduction of non-native disulfide bonds by ERdj5 during the late phase. At the early phase of LDLR translation, non-native disulfide bonds are introduced co-translationally [69,70,71]. At the late phase, these non-native disulfide bonds are isomerized into native disulfide bonds [70,71,72], and ERdj5 probably plays a specific role with BiP in reducing non-native disulfide bonds [72].

Aberrant proteins generated in the ER must be exported into the cytoplasm and degraded by the proteasome via the ER-associated degradation (ERAD) pathway. The reductive unfolding of misfolded proteins facilitates their retrograde translocation via the dislocon channel in the ER, and their subsequent degradation by the ubiquitin-proteasome system in the cytoplasm. The largest PDI family member, ERdj5 (also known as DNAJC10 and J-PDI), reduces aberrant interchain disulfide bonds in the null Hong Kong (NHK) variant of α1-antitrypsin and the J chain of IgM, in concert with immunoglobulin heavy chain binding protein (BiP) and ER degradation-enhancing α-mannosidase-like protein 1 (EDEM1) [67]. The crystal structure revealed that the N-terminus of ERdj5 from *Mus musculus* has a J-domain, of which the general role is to stimulate ATP hydrolysis in Hsp70 family members, and two clusters consisting of Trx-like domains (the N-terminal cluster including Trx1, Trxb1, Trxb2, and Trx2, and the C-terminal cluster including Trx3 and Trx4; Figure 6A,B) [68]. Four domains (Trx1, Trx2, Trx3, and Trx4) contain a Cys-X-X-Cys motif. The redox potential of these active sites is much more reductive than the environment of the ER, and is expected to act as a reductase [68]. Consistently, among the four domains, Trx3 and Trx4 in the C-terminal cluster specifically promote disulfide reduction and the ERAD of NHK in cultured cells [68].

In this NHK ERAD pathway, disulfide-linked NHK oligomers are trapped by EDEM1, which associates with the C-terminal cluster of ERdj5, and aberrant disulfide bonds of NHK are reduced by the catalytic sites of the C-terminal cluster of ERdj5 [68] (Figure 6C). The reduced polypeptide chain of NHK is then transferred to BiP, one of the Hsp70 family members. BiP associates with the His-Pro-Asp (HPD) motif in the N-terminal J-domain of ERdj5 in an ATP-dependent manner [67,68,72,73] (Figure 6B). Finally, BiP dissociates from ERdj5 and transfers the NHK to the retrograde translocation channel to be degraded by the proteasome in the cytosol. These clusters are connected via a flexible linker [68]. Recently, a new crystal structure (form II) suggested a large conformational change between the two clusters compared with the previously solved structure (form I) (Figure 6D). Rotation of Ser557 significantly alters the relative positions of the two clusters (Figure 6E), and HS-AFM analysis of ERdj5 revealed the presence of multiple cluster orientations, and the highly dynamic nature of the C-terminal cluster relative to the N-terminal cluster depending on Ser557 [68,74].

### 4.2. Physiological Roles of ERdj5 in Cells

The dynamics between the N- and C-terminal clusters of ERdj5 are likely involved in client transfer with EDEM1 and BiP along the ERAD pathway. Interestingly, the ERdj5 Ser557Pro mutant, fixed in form I and lacking cluster dynamics, displayed insulin reduction activity similar to that of the wild-type enzyme, but it was less efficient for intermolecular disulfide cleavage of the oligomeric J chain than the wild-type enzyme [74]. Transient expression of Ser557Pro or Gly103Cys/Trp587Cys mutants, lacking cluster dynamics, increased the accumulation of J chain oligomers in cultured cells in the presence of endogenous ERdj5 (wild-type), BiP, and EDEM1 [74]. These results suggest that the cluster dynamics of ERdj5 play crucial roles in the efficient cleavage of intermolecular disulfide bonds in oligomeric misfolded proteins. Only when ERdj5 adopts a form II orientation are its J-domain and Trx3 in close proximity to each other [74], which probably favors substrate delivery to BiP, hence the highly dynamic nature of ERdj5 is also closely related to cooperation with BiP and the handling of misfolded proteins.

ERdj5 is also reported to catalyze the oxidative folding of the low-density lipoprotein receptor (LDLR) [72]. Because the LDLR folding intermediate possesses inter-domain non-native disulfide bonds in the ER [69,70], ERdj5 plays a specific role in reducing non-native disulfide bonds of LDLR to ensure its native folding and domain assembly. Noticeably, inhibition of BiP binding using the ERdj5 His63Gln mutant that does not interact with BiP compromises oxidative folding of LDLR in cultured cells [72]. Recently, optimized two-dimensional gel electrophoresis showed that the inter-domain non-native disulfide bonds of LDLR are generated during the early phase of translation, and translation of the β-propeller of LDLR triggers disulfide shuffling [70] (Figure 6F). These findings strongly suggest that ERdj5 and BiP interact with the entangled polypeptide chain of LDLR containing non-native disulfide bonds to reduce them during the late phase of LDLR translation (Figure 6F).

In addition to ERdj5, other chaperones such as ERp57, ERp72, and CNX were shown to bind to LDLR as it passes through the ER [75,76,77]. Moreover, the binding of misfolded LDLR to SEL1L [75], one of the ERAD components, and the interaction of ERdj5 with SEL1L to promote retrograde transport [78], suggest that ERdj5 is also involved in the degradation of LDLR. Thus, interplay between ERdj5 and BiP seems essential for efficient catalysis of oxidative folding, as well as reductive unfolding and ERAD. As mentioned above, target substrates of ERdj5 and other members of the PDI family are diverse in size, shape, and the number of disulfide bonds. The structural dynamics of the N- and C-terminal clusters likely serve to facilitate the reduction of non-native disulfides and the binding/release of such diverse substrates by ERdj5.

## 5. PDI Family Members Function as Protein Assemblies

### 5.1. PDI and the Microsomal Triglyceride Transfer Protein (MTP) α-Subunit Form a Functional Molecular Complex

**Figure 7:**
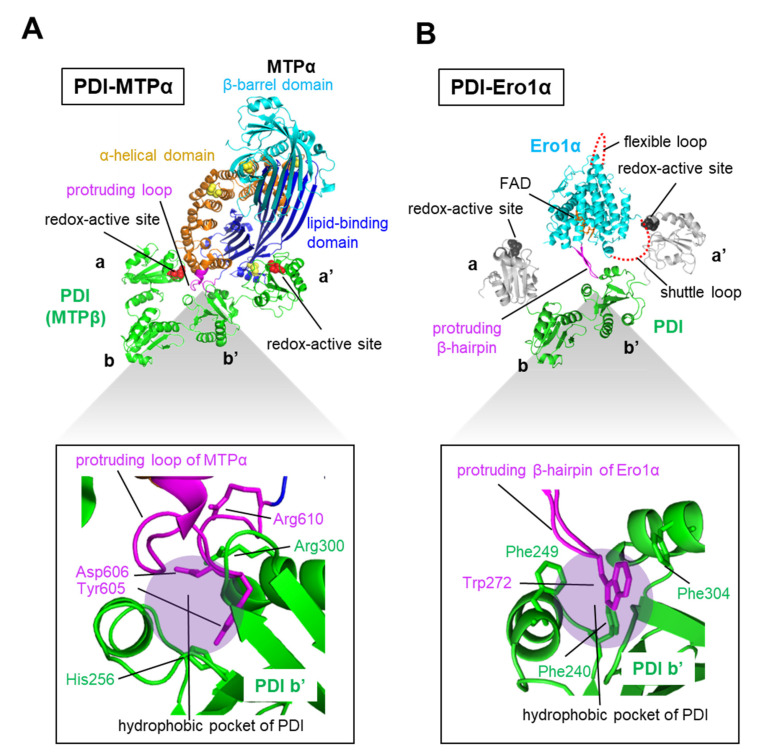
The protruding aromatic ring of *human* microsomal triglyceride transfer protein (MTP)α or *human* Ero1α is in close contact with aromatic or hydrophobic residues in the PDI **b’** domain. (**A**) Crystal structure of the PDI-MTPα complex (PDB code: 6I7S). Upper panel, PDI, β-barrel domain, α-helical domain, and lipid-binding domain of MTPα colored green, cyan, orange, and blue, respectively. Redox-active sites of PDI and disulfide bonds in MTPα are indicated by red and yellow spheres, respectively. Lower panel, interaction sites between the hydrophobic pocket in the **b’** domain of PDI (shadow) and a protruding loop of MTPα (magenta). (**B**) Docking model of the PDI-Ero1α complex predicted by docking simulation analysis of the molecular surface, electrostatic potential, and hydrophobicity complementarity, weighted by the conservation of interacting residues using the sysimm.ifrec.osaka-u.ac.jp/surFit/index.html website. *Human* Ero1α (PDB code: 3AHQ) and the **b**-**b’** domain of *human* PDI (PDB code: 2K18) were docked. The **b**-**b’** domain, Ero1α, flavin adenine dinucleotide (FAD) cofactor, and protruding β-hairpin in Ero1α are colored green, cyan, orange, and magenta, respectively. The putatively positioned catalytic domains (**a** and **a’**) are colored gray. The disulfide bond of the redox-active site in the **a’** domain is formed by catalytic sites on the shuttle loop of Ero1α, which is shown as a red dotted line. The lower panel shows a close-up view of the interaction sites between the hydrophobic pocket in the **b’** domain of PDI (shadow) and the protruding β-hairpin of Ero1α (magenta).

PDI acts not only as a catalyst of oxidative folding and assembly for clients, but also as a functional molecular complex, with proteins such as the β-subunit of microsomal triglyceride transfer protein (MTP) [79] and P4-H [80,81,82]. A recent crystallographic study of a heterodimeric complex comprising an MTP α-subunit and an MTP β-subunit (also known as PDI) revealed that PDI interacts with MTPα via **a**, **b’**, and **a’** domains [79] (Figure 7A, upper). In particular, a protruding loop around amino acids 594 to 610 of *human* MTPα interacts with a hydrophobic pocket in the **b′** domain of PDI. Tyr605 of MTPα plays a major role in this interaction (Figure 7A, lower). Similarly, previous docking models demonstrated that the hydrophobic pocket in the PDI **b’** domain accommodates Trp272 of a protruding hairpin region of *human* ER oxidoreductin-1α (Ero1α) [83,84], a PDI-oxidizing enzyme [85,86,87] (Figure 7B). Thus, the protruding aromatic ring of MTPα or Ero1α is in close contact with aromatic or hydrophobic residues in the PDI **b’** domain. Besides the PDI **b’** domain, the **a** domain interacts with the α-helical domain of MTPα, while the **a’** domain contacts the lipid-binding domain of MTPα (Figure 7A, upper). Notably, disruption of the MTPα and PDI complex leads to aggregation of the MTPα subunit, suggesting that PDI also acts as a chaperone of MTPα. The newly determined complex structure may provide clues to understanding not only the basic principle for MTP subunit assembly, but also the chaperone function of PDI for recognizing aggregation-prone clients.

### 5.2. ERp57-Assisted Supramolecular Peptide Loading Complex (PLC) Assembly during the Loading of Antigenic Peptides into the HLA Heterodimer

**Figure 8:**
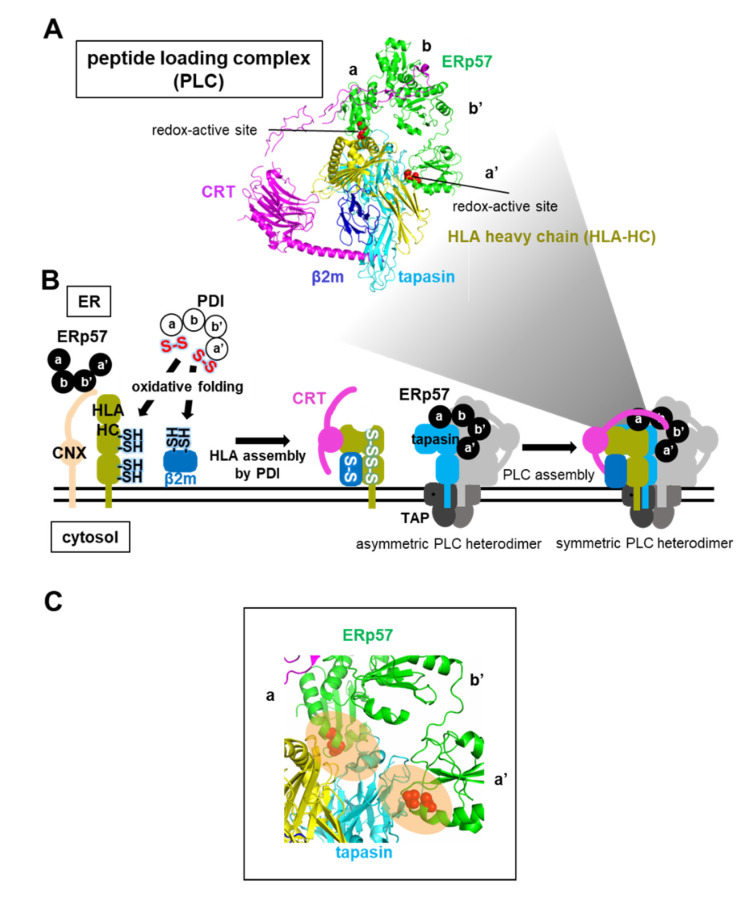
ERp57-assisted HLA and peptide loading complex (PLC) complex formation. (**A**) Cryo-electron microscopy structure of PLC composed of ERp57, CRT, HLA-HC, β2m, and tapasin, colored green, magenta, yellow, blue, and cyan, respectively (PDB code: 6ENY). (**B**) Schematic diagram of the folding and assembly pathway of HLA and PLC. CNX with ERp57 recognizes HLA-HC through monoglucosylated N-linked glycans, and PDI then catalyzes disulfide bond formation of HLA-HC and β2m, and HLA assembly. The HLA heterodimer is recruited to the asymmetric PLC heterodimer by CRT, resulting in the formation of the symmetric PLC heterodimer. (**C**) Close-up view of the complex interface between redox-active sites of **a** and **a’** domains in ERp57 and tapasin (PDB code: 6ENY). Redox-active sites are shown as red spheres.

ERp57 also participates in the assembly of PLC, an immune-related supramolecular complex, in the ER. PLC is a transient multisubunit membrane complex containing HLA, CRT, TAP, ERp57, and the chaperone tapasin [49] (Figure 8A). The HLA heavy chain (HLA-HC) binds to β2m as HLA light chain to form heterodimeric HLA, which acts as a receptor for antigenic peptides to initiate an immune response [88] (see Figure 5B). The reduced form of newly synthesized HLA-HC interacts initially with CNX, and folded HLA-HC containing native disulfide bonds subsequently interacts with CRT [16]. Of note, ERp57 is associated with CRT-HLA or CNX-HLA complexes during all stages of the assembly and folding of HLA. However, knockdown of the ERp57 gene in cells has little effect on oxidative protein folding of HLA-HC and the HLA assembly, while depletion of the PDI gene causes a delay in oxidative folding of HLA-HC and HLA assembly [17]. Taken together, evidence suggests that CNX and ERp57 bind to a nascent HLA-HC, presumably to inhibit aggregation, and PDI then catalyzes the oxidative folding of HLA-HC before β2m association during the early stages of the folding and assembly of HLA (Figure 8B).

The HLA heterodimer without antigenic peptides is recruited by CRT and becomes part of the transient supramolecular PLC, in which the tapasin chaperone stabilizes the HLA heterodimer [49]. ERp57 is also crucial for maintaining the stability of PLC complexes, and for prompting antigenic peptide loading. In addition to the interaction with the arm-like P-domain in CRT (Figure 4C), both **a** and **a’** domains of ERp57 gain access to the N-terminal domain of tapasin [42,49], a fusion of a seven-stranded β-barrel and an immunoglobulin-like domain, through a similar interaction mode to that between MTPα and PDI **a** and **a’** domains [79] (Figure 8C). Consequently, ERp57 helps to maintain the reaction intermediate PLC in a structurally metastable state that can load the peptide into the HLA heterodimer during the latter stages of HLA assembly.

## 6. Conclusions

Recent analysis of ER proteostasis networks has revealed that PDI family members catalyze client folding and domain/subunit assembly through their distinct functions and structures (Table 1). PDI effectively guides the proper oxidative folding and assembly of clients such as hCG, procollagen, plasminogen, and laminin-511 by regulating conformational dynamics and oligomeric states, depending on its redox state, and the configurations or folding states of clients. Unlike PDI, other PDI family members including ERp57 and ERdj5 appear to require functional partners for client folding and assembly. ERp57 catalyzes the folding and assembly of glycoproteins, such as HLA, FcRn, and fibrinogen, in concert with the lectin chaperones CNX and CRT, since ERp57 by itself cannot recruit clients along the client folding pathway, unlike PDI. ERdj5 guides the correct oxidative folding of LDLR in concert with BiP through the reduction of non-native disulfide bonds of LDLR due to the highly dynamic nature of ERdj5. Similarly, the cytosolic chaperonin TRiC also cooperates with a specific holding co-chaperone (prefoldin). After capturing clients, such as actin and tubulin, prefoldin directionally transfers unfolded actin to TRiC for subsequent folding in an effective manner [89]. Therefore, differences in client recognition by enzymes/chaperones may be achieved through the presence of different partners, which allows for control of the folding and assembly of a wide range of clients.

Although client recognition mechanisms of PDI family members can be inferred from the structures determined for MTP [79] and PLC [49], little is known about the detailed mechanism of structural recognition between clients and PDI family members because structures of their complexes along the pathway of client folding and assembly are yet to be determined. Recent advanced hybrid structural approaches using crosslinking mass spectrometry, nuclear magnetic resonance, and cryo-electron microscopy revealed the transient interaction between clients and chaperones/enzymes, such as TRiC [90], a bacterial trigger factor [91], and Hsp40 [92]. Other hybrid approaches, combining not only HS-AFM and SAXS but also HS-AFM and crystal structures, have also provided new insights into the dynamic structural features of client recognition by PDI and ERdj5 [32,74,93]. These common features imply that chaperones/enzymes temporarily hold their clients in a limited hydrophobic space via weak interactions for efficient folding, and the orientations of their subunits and domains are dynamically controlled by client binding and chemical factors such as redox state and ATP. However, structural determination of complexes formed between chaperones/enzymes and their clients remains challenging from a mechanistic perspective. Further advances in structural biology, including combinations of the above-mentioned techniques, will likely assist the determination of such complex structures, and ultimately reveal details of the proteostasis mechanism at the atomic level.

## Figures and Tables

**Table 1:** Protein assembly and disassembly by PDI family members.

PDI Family Members	Substrates	Activities	Location	References
PDI	hCG	Assembly of hCGβ and hCGα	ER	[37]
PDI and ERp57	HLA	Assembly of HLA-HC and β2m	ER	[16,17]
ERp57	FcRn	Assembly of FcRn-HC and β2m	ER	[54]
	Fibrinogen	Assembly of three polypeptides (Aα, Bβ, and γ)	ER	[66]
ERdj5	LDLR	Disassembly by inter-domain disulfide reduction	ER	[72]

## Abbreviations

BiP	immunoglobulin heavy chain binding protein
BPTI	bovine pancreatic trypsin inhibitor
CCT	chaperonin-containing tailless complex polypeptide 1
CNX	calnexin
CRT	calreticulin
EDEM1	endoplasmic reticulum degradation-enhancing α-mannosidase-like protein 1
ER	endoplasmic reticulum
ERAD	ER-associated degradation
ERdj5	endoplasmic reticulum DNA J-domain-containing protein 5
Ero1α	ER oxidoreductin-1α
ERp27	endoplasmic reticulum resident protein 27
ERp57	endoplasmic reticulum resident protein 57
FcRn	neonatal Fc receptor
hCG	human chorionic gonadotropin
HLA	human leukocyte antigen
HS-FM	high-speed atomic force microscopy
IgG	immunoglobulin G
IgM	immunoglobulin M
LDLR	low-density lipoprotein receptor
MHC	major histocompatibility complex
MTP	microsomal triglyceride transfer protein
PDB	protein data bank
PDI	protein disulfide isomerase
PLC	peptide loading complex
P4-H	prolyl 4-hydroxylase
RNase A	ribonuclease A
SAXS	small angle X-ray scattering
TAP	transporter associated with antigen processing
TRiC	tailless complex polypeptide 1 ring complex
Trx	thioredoxin
β2m	β_2_-microguloblin
